# Grandmother effects over the Finnish demographic transition

**DOI:** 10.1017/ehs.2023.36

**Published:** 2024-01-04

**Authors:** Simon N. Chapman, Virpi Lummaa

**Affiliations:** 1 INVEST Research Flagship Centre, University of Turku, Finland; 2 Department of Biology, University of Turku, Finland

**Keywords:** Longevity, survival, grandmother hypothesis, demography, fertility

## Abstract

Demographic transitions are defining events for human societies, marking shifts from natural mortality and fertility rates to the low rates seen in industrialised populations. These transitions can affect trait evolution through altering the direction and strength of selection when variance in fertility and mortality decline. One key feature of human evolution is the evolution of extended post-reproductive life through indirect fitness benefits from grandmothering. Although studies in pre- and post-transition societies have documented beneficial grandmother presence, it remains unknown whether these associations changed before, during, or after the transition. Here, we use genealogical data from eighteenth- to twientieth-century Finland to show grandmother-associated changes of two measures of evolutionary fitness (grandchild survival and birth rate) over the transition. We find that grandmothers had greater opportunity to help as the transition progressed, but their effect on grandchild survival declined alongside general mortality rates, implying that selection on lifespan from grandmothering declined too. Whilst grandmother presence was still associated with reduced birth intervals and hence more grandchildren born post-transition, the nature of this relationship changed greatly. This suggests that although potential for intergenerational interactions increased over the demographic transition, the (hypothesised) evolutionary importance of these interactions declined, which reduced selection for extended post-reproductive lifespan.

## Graphical abstract

**Figure:**
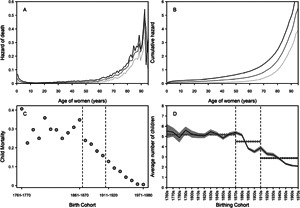

**Social media summary:** Church registers show that the demographic transition in Finland changed the role of grandmothers

## Introduction

Kin selection theory posits that evolutionary fitness can be improved directly via one's own reproduction, or indirectly from helping kin (Hamilton, 1964). There is extensive empirical evidence in the literature from a wide variety of species, including humans, where kin-associated beneficial outcomes have been found across different societies and in different cultural contexts (Sear & Mace, 2008). One trait proposed to have evolved through such kin interactions is extended post-reproductive lifespan. Extended post-reproductive lifespan is rare in nature, limited to humans and a small selection of toothed whales (Ellis et al., 2018a, b). In these species, the presence of post-reproductive females can increase the fertility of offspring or improve the survival of grandchildren, thereby increasing the evolutionary fitness of long-lived individuals even beyond the point when they can no longer produce their own offspring (menopause). Such indirect fitness benefits from grandmothering (Sear & Mace, 2008; Hawkes et al., 1998; Lahdenperä et al., 2004; Chapman et al., 2019) may have played a key role in the evolution of extended post-reproductive lifespan in these species (Hawkes et al., 1998).

However, selection from grandmothering on lifespan in humans has been limited by mortality constraints in the past (Chapman et al., 2019), with similar patterns of longevity seen across different pre-industrial societies (Chapman et al., 2019; Gurven & Kaplan, 2007). In contemporary industrialised societies, human lifespan is longer than ever before, attributable to societal changes and medical advances (Oeppen & Vaupel, 2002). With the alleviation of mortality constraints on older cohorts, a possible limit to the ability of grandmothers to provide help is lifted: selection may be able to act on longevity further, facilitated by modern medicine and society. However, this increasing opportunity for grandmothers to help is countered by the typical consequence of industrialisation: a demographic transition. Demographic transitions are society-defining, involving a large shift from natural mortality and fertility rates to the low rates seen in contemporary industrialised populations (Bongaarts, 2009; Riley, 2001). A demographic transition can greatly affect the evolution of traits via changing selection pressures from the societal processes that triggered the transition (e.g. alleviation of sources of mortality) and through strength of selection changing with variance in fertility and mortality (Courtiol et al., 2013; Arnold & Wade, 1984; Scranton et al., 2016; Stearns et al., 2010; Corbett et al., 2018) (e.g. mortality outcomes becoming less variable with advances in medicine and hygiene practices). Critically, despite documentation of the benefits of grandmother presence in many pre- and post-transition societies (Sear & Mace, 2008; Sear & Coall 2011), we do not know whether these associations changed during the transition, or much later, after the major fertility and mortality changes had already occurred.

Whilst child mortality had a higher variance and therefore a higher potential strength of selection in the pre-industrial past, the demographic transition reduced this to negligible levels (Scranton et al., 2016). The role of grandmothers as vital life-preservers for children (Sear & Mace, 2008) is therefore likely to have diminished rapidly over the demographic transition as the need for survival-boosting help declined. However, even though less often considered (Chapman et al., 2021), the grandmother hypothesis is not solely concerned with increasing grandchild survival, but also with increasing the number of grandchildren produced by the middle generation (Hawkes et al., 1998). While fertility also decreased during the demographic transition (Bongaarts, 2009; Scranton et al., 2016), natural selection can continue to operate on humans as long as variance in fitness is still greater than zero (Courtiol et al., 2013; Scranton et al., 2016; Stearns et al., 2010; Corbett et al., 2018). Understanding how the demographic transition from high mortality and high fertility to low mortality and low fertility affects the realised benefits of grandmothering, and whether grandmothers retain an evolutionary role in more industrialised societies, is therefore crucial for gaining further insight into the importance of grandmothering as a contributor to the evolution of extended post-reproductive life.

Here, we use genealogical records from eighteenth to twentieth century Finland to establish how the opportunity and need within the grandmother–grandchild relationship changed across and beyond the demographic transition. In Finland, the demographic transition occurred relatively late, starting in the late nineteenth century and lasting into the early twentieth century (Scranton et al., 2016). Our data provide a rare opportunity to measure grandmother effects on survival and fertility longitudinally in the same population across the different stages of the demographic transition, in order to understand how modernity transformed selection on the key human life-history trait of extended post-reproductive lifespan.

## Methods

### Study population

For this study, we used extensive genealogical records from Finland. These records come from two main sources: Lutheran parish registers and published genealogies. The former were collected by Lutheran priests – intensively from 1749 as mandated by law – and cover major events such as births, deaths and marriages. From these, individual life-histories and lineages can be reconstructed.

While registers are available for the entirety of Finland, as required by the Swedish Church Law of 1749, most have not been digitised and linked across different types of records and across generations. As such, our study includes lineages originating in eight parishes across four regions of Finland whose marriage, birth and death records have been digitised and linked from early 1700 until, in some cases, the present day (Southwest Finland: Rymättylä, Hiittinen, Kustavi; Pirkanmaa: Ikaalinen, Tyrvää; Northern Ostrobothnia: Pulkkila; Karelia: Rautu, Jaakkima). Although slight geographic variation exists across the country in birth and death rates, this subset covers different regions of Finland, including those using different farming methods (Moring, 1993, 1999) and different household compositions (Moring, 2003), and with differences in mortality (Moring, 1998), and can therefore be considered as broadly representative of the whole population.

Pre-industrial Finland was highly agrarian, with arable cultivation the predominant method (Holopainen & Helama, 2009) with slash-and-burn more common in the more heavily forested eastern regions. Households were often large, as the labour requirements for farms often exceeded the capabilities of a basic nuclear family unit (Moring, 1993). Despite a heavy reliance on crops (Vihola, 1994), agricultural techniques and innovations were limited (Holopainen & Helama, 2009), and localised famines were a recurring threat. Industrialisation began in the 1860s, later than elsewhere in Europe (Hjerppe, 1989), and was shortly followed by the onset of the demographic transition in the 1870s (Scranton et al., 2016). In this final pre-transition decade, the first railway was opened (1862) (Kotavaara et al., 2011). In the years following the onset of the transition, population vital rates continued to improve (Scranton et al., 2016), restrictions on the division of land were relaxed and nuclear households increased in frequency (Moring, 1993). Mobility increased greatly as time progressed, particularly in post-World War II Finland, where rapid motorisation was accompanied by population shifts to urban areas (Kotavaara et al., 2011).

### Statistical analysis

We conducted discrete time-event analyses using binomial generalised linear mixed-effect models with a logit link. These were implemented with *lme4* version 1.1-23 (Bates et al., 2015) in R version 4.0.3 (R Core Team 2020). The significance of interactions was evaluated using likelihood ratio tests with the function *mixed* from the package *afex* version 0.28-0 (Singmann et al., 2017). Significance was assessed at the level of *α* = 0.05. Based on Scranton et al. (2016), we defined three time periods as: pre-industrial, 1761–1870; transitional, 1871–1910; and post-transition, 1911–1980. We ran all models again with slight alterations to the start and end points of the transitional period to test the robustness of the period selection to slight variation in years (see Table S1 and associated text in the supplementary information). From the consolidation of these checks, we feel that the selected cut-off (1871–1910) is appropriate and does not affect the conclusions.

One limitation of historical register data is that causality is much more challenging to establish – we emphasise that the results shown here are not causal and are instead associations. Factors that may influence helping behaviour such as co-residence or age and frailty of grandmothers were not accounted for in this study owing to limitations of sample sizes over the time period. While these can play some role in the associations between grandmothers and fitness outcomes (Chapman et al., 2019, 2023; Hacker et al., 2021; Willführ et al., 2022), these specific factors are unlikely to bias the results of the present study, as they mostly affect paternal kin (Chapman et al., 2019, 2023), particularly co-residence, which was predominantly patrilocal (Chapman et al., 2023). There may be other unobserved factors that we could not account for, and as such we ask readers to keep this caveat in mind.

### Hazard functions

To determine age-specific instantaneous hazard rates of women in the three time periods (pre-industrial, transitional and post-transition), we obtained Kaplan–Meier type hazard estimates using the *kphaz* function with the Nelson–Aalen estimator from the package *muhaz* version 1.2.6.4 (Hess & Gentleman, 2021). This function accounts for right-censoring. For the pre-industrial hazard function, we included all individuals alive at any point between 1761 and 1870 (*n =* 19270). Those born in this period but with a last record (either censored or dying) after 1870 were censored at 1870, regardless of actual date of death or censoring. This was done to ensure that hazard rates were not influenced by the introduction of societal improvements that could affect survival after the time period. For the transitional hazard function, we included all of those alive at any point during 1871–1910 (*n =* 25,993), again censoring those with a last record after 1910 at 1910. For the post-transitional period, we included all those alive from 1911 and born before 1981 (*n =* 18,852). As such, an individual could be present in all three periods, albeit with different survival times; for example, an individual born in 1860 and dying in1933 would be considered as age 10 (censored) pre-industrially, age 50 (censored) transitionally, and age 73 (died) post-transitionally. For robustness purposes, we ran the models again without censoring at the end of the pre-industrial and transitional periods (Figure S1), but our conclusions did not change. To obtain the cumulative hazard functions, we used the *cumsum* function on the hazards from the *kphaz* models.

### Grandchild survival

To analyse the effect of grandmothers on survival, we selected individuals born between 1761 and 1980. In Finland, grandmother effects on survival are context-dependent, with beneficial associations with grandchild survival only found for toddlers (ages 2–5) and only if there was a nearby maternal grandmother (Chapman et al., 2019, 2021; Nenko et al., 2021). As such, we focus only on the effects of maternal grandmothers (*n =* 4497) on the survival of 2- to 5-year-old grandchildren (*n =* 27,204) (i.e. the age and lineage combination that has been shown to be beneficial in the pre-industrial era). We additionally ran the models again with paternal rather than maternal grandmothers, in order to check whether they may have exhibited change over time too. In line with our assumption that there would be no grandmother effects present for paternal grandmothers (based on previous work in this population, e.g. Chapman et al., 2021), these models did not indicate associations between paternal grandmother presence and the measures of biological fitness, nor did they suggest changes in these (non-)associations over time. As such, these results are not reported in the main text, but can be found in the Supplementary Information.

Geographic distance is important regarding opportunity for grandmothers to be able to help (Engelhardt et al., 2019). As housing information is unavailable in the digitised records for the majority of individuals in our dataset, geographic distance between grandchild and grandmother was instead proxied on a broad-scale by comparing the birth parish of a grandchild with the last known parish of a grandmother. If a grandmother was coded as ‘alive’ and present in the analysis, she needed to have lived in the same parish as the grandchild or a neighbouring parish. Those grandmothers living further afield were not included, as their inclusion may mask possible associations between grandmother presence and grandchild survival.

The response variable was survival in a given year, coded as a binary 0 for dying in that year and 1 if the focal individual survived. Our main explanatory variable was the interaction of time period (three-level factor: pre-industrial, transitional and post-transition) and maternal grandmother status (two-level factor: no living maternal grandmother and maternal grandmother present) to test whether the association between grandmother presence and child survival changed through time. In a separate model, we used birth cohort (coded as continuous) instead of time period in the interaction, in order to get a finer-scale view of the changes. However, we advise some caution in the interpretation of this latter model: sample sizes in the later cohorts are much lower than in the nineteenth to early twentieth century cohorts, which may affect estimates.

We included age of the child (continuous), mother survival status, number of living siblings under the age of 18, sex, childhood social class (based on the occupation of the father; two-level factor – landed or landless) and twinning status (not a twin or twin) as additional fixed effects to account for their potential association with child survival. Number of living maternal cousins was included to account for dilution of grandmother effects from potentially competing cousins. Mother ID nested within grandmother ID was included as a random effect, to account for shared family at the sibling (mother) and cousin (grandmother) level. Birth cohort (22-level factor) was included as a random effect only in models where the broad ‘time period’ fixed effect was used.

To avoid overfitting models (see Burnham & Anderson, 2002), we calculated Akaike information criterion (AIC) values for models sequentially excluding each term. We retained terms in the final model if the AIC value of a model excluding that term was more than two higher than the base model. Sex and childhood social class were not included in the final survival models following this AIC approach. Violin plots were created with *vioplot* from the *vioplot* R package version 0.3.7 (Adler & Thomas Kelly, 2021).

### Fertility analyses

As fertility cannot adequately be described with only a single measure, we separately investigated whether grandmother presence was associated with two components of fertility – age at first birth and inter-birth intervals – and whether any associations changed across the transition.

First, we investigated age at first birth. We included women from age 16 up to their first birth or until age 50 (*n =* 2119). Women were only included if they were married and had known information on the mother-in-law, to enable us to see the relative influence of mother vs. mother-in-law. Individuals were censored if their first spouse died before they gave birth to their first child, as this would disrupt scheduling and affect their age-specific marriage probabilities.

The response variable was whether the focal individual had their first birth in a given year (0 = no birth, 1 = birth). Our main explanatory variable was a three-way interaction of age with survival status of the mother/mother-in-law (time-varying four-level factor – only mother alive, only mother-in-law alive, both alive, neither alive) and time period (3-level factor – pre-industrial, transition, post-transition). We ran an additional analysis with birthing cohort (decade of reproduction) replacing time period, similar to the survival analysis above, but the conclusions did not differ.

Following Chapman et al. (2021), we set fixed effects as a quadratic age term, birth order of the focal individual and number of reproductive sisters, to account for possible dilution of the effect of mothers with more reproductive-aged daughters. Mother ID was entered as a random effect to account for possible shared family effects between siblings. Birthing cohort was not included as a random effect when the interaction contained a time period term, as average age at first birth did not vary across the demographic transition (Chapman et al., 2018).

The second set of fertility analyses concerned birth spacing. We included only those women who had given birth more than once, with observations starting from the year after the birth of their first child (*n =* 2047). The response variable was whether the focal individual gave birth in the focal year (1 = birth, 0 = no birth).

Our main explanatory variable was a three-way interaction between time period, survival status of the mother/mother-in-law and time since last birth (time-varying continuous). Time since last birth was preferred to inter-birth interval as it can vary with time and allows retention of individuals in the model after their last birth. Here, we diverged from the modelling structure of Chapman et al. (2021), as a four-way interaction (with age) is difficult to both interpret and visualise. All two-way interactions were included. Unlike the other analyses, we did not run an analysis substituting time period for birth(ing) cohort, as we did not have a sufficient spread of data across the cohorts.

We included birth order as a fixed effect. This birth order term was not of the focal individual, but for the child born at the end of the interval (e.g. all years between the birth of the second and third children – including the birth year of the third child – would be given a birth order value of 3), and was included because parity could affect the desire to have more children. After the final birth, additional years up to age 50 were coded as the next value in the birth order sequence (e.g. if last birth was the third child, additional years would have a birth order set as 4). The survival status of a previous child can also influence birth spacing (Sear et al., 2003), so we included an interaction of previous child survival (time-varying binary; alive = 1, dead = 0). Observations were not removed if time since last birth exceeded 10 years (see Mace & Alvergne, 2012) – instead, they were capped at 10 years to prevent results being biased by possible extreme values (e.g. from those finishing reproduction early) (Chapman et al., 2021). In addition, the number of reproductive sisters was added in this analysis too, also to account for possible dilution of effects, e.g. if the mother spreads her investment across all reproductive daughters.

Individual ID was nested within mother ID as a random effect to account for individual characteristics that could influence inter-birth intervals (as each individual could experience multiple events [births]) and to account for possible shared family effects with siblings.

## Results

**Figure 1.**
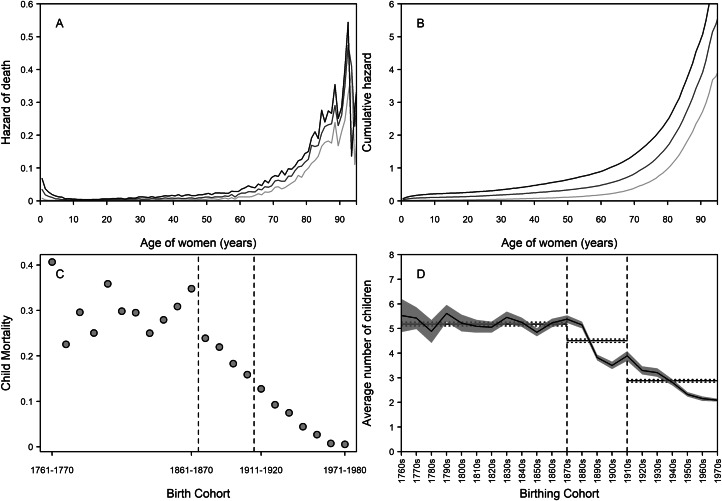
Demographic changes in Finland from 1761 to 1980 in longevity, childhood mortality, and fertility. (a) Hazard functions for survival of women born in pre-industrial (dark grey), transitional (medium grey), and post-transition (light grey) periods. On the left side of the figure, it can be seen that early-life hazard of death became lower. At older ages, the hazards increased greatly in all time periods, but started rising earliest in the pre-industrial period and latest in the post-transitional period (see also Figure S1). (b) Cumulative hazard functions for survival of women in pre-industrial (dark grey), transitional (medium grey), and post-transition (light grey) periods. Risk of death accumulated fastest in the pre-industrial period – we would expect that a woman living in this period would die earlier than in the other periods. (c) Decennial early childhood mortality rates. Following the onset of the transition, mortality rates decline continuously to negligible levels by the later twentieth century. (d) Mean number of children by birthing cohort (95% confidence intervals shown with the shaded area). Over the pre-industrial period, family sizes were larger, with over five children on average. During the transition, average family sizes declined, and continued to do so to the end of the study period. Horizontal lines in each part of (d) are the mean and 95% confidence intervals of number of children per family across each period. Dashed lines in (c) and (d) demarcate the beginning and end of the transition (1871–1910).

Quantifying the demographics of the grandmother–grandchild relationship is important for getting a broad-scale indication of the upper limits of interactions (Margolis, 2016; Margolis & Wright, 2017; Leopold & Skopek, 2015; Chapman et al., 2017, 2018) and therefore on limits to possible selection. Previous work has established that, on average, grandmothers and grandchildren in this population had less than 10 years of shared time pre-industrially, with a large increase in this time following the demographic transition (Chapman et al., 2018) – in other words, grandmothers had a greater opportunity to help over time. This is probably due to both higher longevity (Figure 1a and b) and declining childhood mortality (Figure 1c). Figure 1a and b show the hazard of death and cumulative hazard of death for women living in the pre-industrial (1761–1870), transitional (1871–1910) and post-transition (1911 to present) periods. As Finland progressed to modernity, instantaneous hazard rates at young ages declined. Accelerations in hazards with age indicate increasing senescence and a declining force of selection on lifespan – from these figures, we can see that lifespan was expected to be shorter in the pre-industrial period as hazards start to rise at earlier ages than in the other periods. This acceleration occurs later in the post-transition period, suggestive of (slightly) later onset of senescence and therefore increased longevity.

### Declining importance of grandmothers for grandchild survival

For the grandmother hypothesis, child mortality rates are one of the most important indicators of need. In the pre-industrial period, the child mortality rate was typically high, and in Finland 30.2% of the population died before the age of five during 1761–1870 (Figure 1c). Although there was considerable variation in mortality between birth cohorts, all had under-five mortality rates of over 20%. The mortality in the transitional period (1871–1910) was almost a third lower, at 19.3%, and there was a continuous decline in mortality rate with each passing decade. Post-transition (1911–1980), pre-five childhood mortality was at 6.6%, though again there was a continual decrease to the point that less than 1% of children died before the age of five in the 1960s and 1970s, and the majority of these deaths occurring in early infancy. Hence, the need for kin help to ensure child survival seemingly collapsed over the transition.

**Figure 2.**
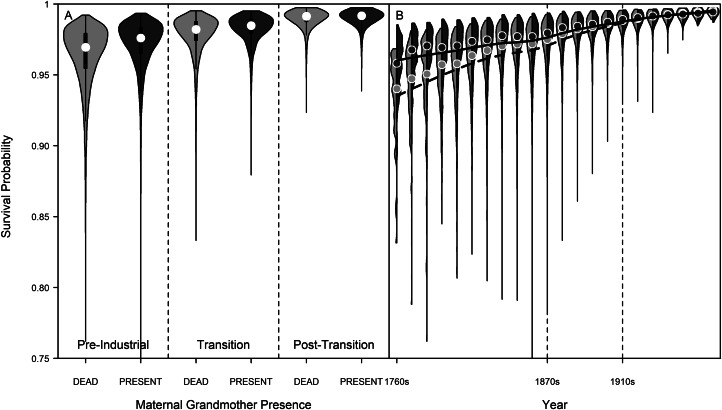
Violin plots of model-predicted survival probabilities for grandchildren by maternal grandmother presence and birth cohort. Vertical dashed lines at 1870s and 1910s demarcate the beginning and end of the demographic transition. Points show the median predicted survival values, and shaded areas show the density distribution of these survival values. Dark grey represents survival values when the maternal grandmother was present, light grey when she was dead. Bandwidths were set as 0.003. (a) Violin plots by time period and grandmother presence. (b) Violin plots by grandchild birth cohort and grandmother presence. Smoothing splines fitted from model predictions using *smooth.spline* function with 8 degrees of freedom, with a solid black line for maternal grandmother present and dashed black line for maternal grandmother deceased. Together, these panels show there was an association between grandmother presence and grandchild survival in the pre-industrial period, but that this association declined over the transition as baseline survival increased. By the end of the demographic transition, maternal grandmothers were no longer associated with improved survival of toddlers. See also Table S2.

The association between grandmother presence and the fitness outcomes of grandchild survival similarly changed over the transition (time period interaction: *χ*_2_^2^ = 9.69, *p* = 0.010; Figure 2a). While survival outcomes for grandchildren were higher pre-industrially when their maternal grandmother was present (*β* = 0.514 ± 0.209, *p =* 0.014), this association was no longer present in the transition period (*β* = 0.308 ± 0.209, *p =* 0.141; but see Supplementary Information), and was absent again post-transition (*β* = −0.228 ± 0.200, *p =* 0.254). The birth cohort interaction with maternal grandmother presence was significant (*χ*_1_^2^ = 4.27, *p =* 0.041; see Methods for caveats), Figure 2b shows a trend of grandchild survival being higher with a maternal grandmother present (cf. dead) across the pre-industrial and early transitional cohorts, before converging by the turn of the twentieth century, resulting in little to no difference in survival by maternal grandmother presence. We note that due to a low number of events in the post-transitional period (i.e. very few deaths of young children), the missing effect of grandmothers in this period could, in part, be driven by sample size and that an effect of some kind may still be present. In this particular case, however, the number of deaths is itself informative of the decreasing childhood mortality and declining need for grandmother's help for grandchild survival.

### Changes in the associations of grandmothers with offspring fertility

Concomitantly with the mortality decline, fertility also declined (Figure 1d), reducing from an average family size of 5.53 ± 3.65 (mean ± standard deviation) children in the 1760s to 2.15 ± 1.11 children by the 1970s. At the start and end of the demographic transition, the average number of children changed from 5.38 ± 3.06 (1870s) to 3.50 ± 2.60 (1900s). Importantly for natural selection, the variance in fertility between Finnish women greatly increased during the period (Scranton et al., 2016), opening opportunities for grandmothers to improve their post-reproductive fitness through boosting offspring fertility. Here, we used generalised linear mixed-effect models to assess whether the demographic transition changed the association of (grand)mothers with beneficial outcomes to fertility traits.

Age at first birth did not significantly differ by mother/mother-in-law presence even in the pre-industrial era (see also Chapman et al., 2021), and there was no significant change in the age-specific probabilities of first birth across the demographic transition with mother/mother-in-law presence either (time period interaction: *χ*_6_^2^ = 3.68, *p =* 0.720; birthing cohort interaction: *χ*_3_^2^ = 1.07, *p =* 0.783; Table S3).

**Figure 3.**
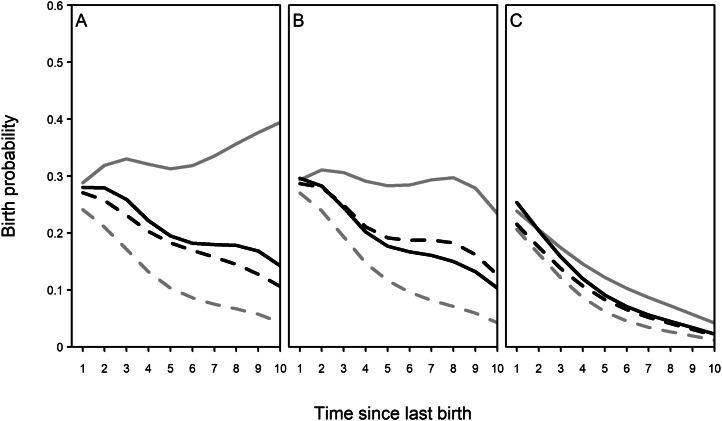
Model-predicted probabilities of subsequent reproduction by mother presence, time since last birth, and time period. (a) Pre-industrial era, (b) transitional period, (c) post-transition period. Line colour and type indicate mother/mother-in-law combination: solid grey = both alive; dashed grey = both dead; solid black = mother only; dashed black = mother-in-law only. Mother and mother-in-law presence associated with higher probabilities of reproduction (i.e. shorter birth intervals) when both were alive. Smoothing splines fitted from model predictions using *smooth.splines* function, with 5 degrees of freedom. See also Table S3.

Birth spacing is known to have been associated with mother/mother-in-law presence in the pre-industrial era in Finland (Chapman et al., 2021). Here, we found that though this association changed over time (time period interaction: *χ*_6_^2^ = 14.09, *p =* 0.029), birth spacing remains associated with both mother/mother-in-law presence at the same time (Figure 3; Table S3). Across all periods, having both mother and mother-in-law alive was associated with the highest probabilities of birth, although only mothers or only mothers-in-law had no significant effect.

## Discussion

The demographic transition in Finland brought about large changes in mortality and fertility. As expected, female hazard of death pre-industrially was higher in infancy than during or after the transition, indicative of increased childhood mortality. Similarly expected was the increase in lifespan during and especially after the transition (Oeppen & Vaupel, 2002; Bongaarts, 2009; Burger et al., 2012), indicated by a later acceleration in instantaneous hazard rates. Accumulation of risk was such we would expect a woman living in the pre-industrial period to have died by age 62, compared with age 73 in the transitional period and age 80 in the post-transition period. To put it more simply, lifespan increased over and beyond the transition and therefore grandmothering could potentially have continued for longer. There was, therefore, greater opportunity for grandmothers to aid their grandchildren. However, as childhood mortality decreased over and beyond the demographic transition, the need for this kind of grandmother help also declined. Indeed, we found that the beneficial effect of maternal grandmothers on grandchild survival seen pre-industrially was no longer found over the transition or post-transitionally. One possible mechanism could be the role of grandmothers in reducing mortality to diseases that were, pre-industrially, deadly to young children, e.g. smallpox, various pulmonary issues and diarrhoeal infections (Ukonaho et al., 2023), but with improved hygiene and medical care were no longer major causes of mortality in the twentieth century. As such, grandmother help would not be observed in survival outcomes.

There were no effects of mother/mother-in-law presence on age at first birth before, during and after the demographic transition. This is probably due to the cultural importance of marriage and the marriage practices of Finland. Marriage could almost be considered a pre-requisite to reproduction (Pettay et al., 2020), and age at marriage remained fairly stable, at least in the pre-industrial period (Moring, 1996). However, the other aspect of fertility studied here, birth spacing, was influenced by the presence of the elder generation. Mother-in-law influence on fertility is not uncommon in other societies (Sear et al., 2003; Tymicki, 2004; Tanskanen et al., 2014), even in the absence of any beneficial effect of paternal grandmothers on grandchild survival (Sear et al., 2002), and this appears to be the case in Finland too. While the presence of both mother and mother-in-law associated with higher probabilities of subsequent birth before, during and after the transition, the presence of mother or mother-in-law alone was not significantly associated with higher subsequent birth probabilities. Therefore, although grandmother presence does seem to still be linked to reduced birth spacing among daughters and sons and therefore potential for more grandchildren, the nature of family networks and the effects thereof changed too.

Although the Finnish demographic transition is fairly typical in the pattern of mortality and fertility change (Scranton et al., 2016), we urge caution in generalising the results here to changing effects of grandmother presence across demographic transitions in other societies. Benefits associated with grandmothering are not entirely consistent across societies (Sear & Mace, 2008; Sear & Coall, 2011), and can depend on various contexts, e.g. lineage (maternal or paternal) (Chapman et al., 2021; Sear et al., 2002; Jamison et al., 2002; Sheppard & Sear, 2016), sex (Fox et al., 2010; Tanskanen et al., 2011; Tanskanen & Danielsbacka, 2021), age (Chapman et al., 2019), distance (Engelhardt et al., 2019), birth status (Nenko et al., 2021), co-residence (Hacker et al., 2021; Willführ et al., 2022; Chapman et al., 2023), and perhaps most importantly, differences in culture (Chapman et al., 2021; Coall & Hertwig, 2010). As such, the exact patterns of change over demographic transitions may vary between societies, and we urge replication in other suitable populations (i.e. post-transitional with available historical records). However, it is almost certainly the case that, in a broad sense, opportunity for grandmother help increased – as demographic transitions have decreased childhood mortality and lifespans have increased (Riley, 2001) – and the need for fitness-related help declined for much the same reason. This is not to say that grandmother help ceased or even simply declined, just that the nature of the help provided no longer affected grandchild survival outcomes. For example, monetary transfers or supplemental childcare from grandmothers can be greatly beneficial to families without having or needing observable evolutionary outcomes.

We have shown that grandmothers had greater opportunity to help as the transition progressed, but that their effect on grandchild survival declined as general mortality rates declined, implying that selection on lifespan from grandmothers’ child mortality-reducing behaviours declined too. Although grandmother presence was still linked to reduction in birth intervals post-transition, the nature of this relationship changed greatly. These changes suggest that though the demographic transition increased the potential for interactions between generations, it was also associated with a decline in the (hypothesised) evolutionary importance of these particular interactions. Selection from other aspects of grandmothering can still persist, especially in regards to reproductive success, so this does not mean grandmothering has been decoupled from evolutionary selection on lifespan. These results add to the increasing body of evidence showing how the transition to modernity has altered the direction and intensity of natural selection acting on many traits (Courtiol et al., 2013; Scranton et al., 2016; Stearns et al., 2010), with important implications for public and global health (Corbett et al., 2018). Current demographic pressures – including decreasing fertility, increasing life expectancy and increased migration and urbanisation – are further altering the structure and dynamics of our family structures and social networks. Understanding the role of changing social networks in determining the timing and number of children, intergenerational relations, and active ageing is therefore of key importance for developing better social and health policies for the twenty-first century.

## Supporting information

Chapman and Lummaa supplementary material 1 Chapman and Lummaa supplementary material

Chapman and Lummaa supplementary material 2 Chapman and Lummaa supplementary material

Chapman and Lummaa supplementary material 3 Chapman and Lummaa supplementary material

## Supplementary material

To view supplementary material for this article, please visit https://doi.org/10.1017/ehs.2023.36

## Research transparency

Data and code used in this work are available as supplementary material.
