# Supplementary material for: Grandmother effects over the Finnish demographic transition
Source: Evol Hum Sci. 2024 Jan 4;6:e6. doi: 10.1017/ehs.2023.36 (PMC10955376; doi:10.1017/ehs.2023.36)
Supplement: Chapman and Lummaa supplementary material 2 — Chapman and Lummaa supplementary material [file S2513843X23000361sup002.docx]

**Robustness of demographic transition cut-offs**

To check the robustness of our results to the cut-offs used for time period, we ran the analyses again with the start and end points of the transitional period increased by 1, 3, 5, and 10 years in three different ways: changing the start of the transitional period only (end point remains 1910), changing the end of the transitional period only (start point remains 1871), and by changing both start and end points of the transitional period (Table S1).

Whilst shifting the start of the transitional period by one year is the ‘best’ model according to Akaike Information Criterion (AIC) for age at first birth, the ∆AIC from the cut-offs we used in the papers is only 4, suggesting that the models are not greatly improving the fit to the data. Indeed, it is somewhat of a moot point for this analysis anyway, as the interaction remains non-significant (i.e. our conclusions are the same about no change over time in regards to the non-association of mother/mother-in-law presence with age at first birth).

The situation is similar in regards to birth spacing, with a significant three-way interaction in all cases, and the visualisation of the interaction also looking similar. Actual model estimates varied slightly depending on the cut-off, but the main conclusion was always the same as reported in the paper: both mother and mother-in-law presence was most important for shortening birth intervals. Shifting the start of the transitional period by one, three, or five years showed a significant positive effect of the mother only (i.e. mother-in-law dead; one year β = 0.121±0.058, p = 0.036; three years β = 0.118±0.058, p = 0.041; five years β = 0.115±0.058, p = 0.048), with a similar effect size to the insignificant effect in the reported model (β = 0.111±0.057, p = 0.054). AIC values for these models were, however, larger than for the base model. The fertility analyses, therefore, appear robust to the choice of cut-off points for the start and end of the demographic transition.

In the survival analysis, the interaction of grandmother presence and time period remained significant in all cases, indicating the broad conclusion of a declining need for help in regards to survival held regardless of whether the transition was extended or not. However, whether grandmother presence significantly associated with survival across the transition was somewhat sensitive to the length of the transition period, with maternal grandmother presence significantly associating with increased grandchild survival when the transition end point was 3, 5, or 10 years later, and when the beginning and end points were 3, 5, and 10 years off the reported transition. The AIC was only lower than the reported model for three of the models though, with the lowest AIC value for the 1870-1910 transition (cf. 1871-1910), which had very similar coefficients to the reported model. The 1870-1911 transition also had similar coefficients to the base model. For the other model with a lower AIC than the reported model - the 1871-1920 transition – grandmother presence was significantly associated with higher survival over the transition period (β = 0.652±0.316, p = 0.039; cf. base model β = 0.308±0.209, p = 0.141).

For the hazard of death of women, the conclusions drawn from the cumulative hazard function do not change either when shifting the start of the transition to being one year earlier – the expected age that a woman would die remains as reported on line 343 – 62 pre-industrially, 73 over the transition, and 80 post-transition. None of the time-period shifts affect this age 62 for pre-industrial era, and post-transition age at expected death only rises by one year to 81 if the transition is extended for 10 years. The age at expected death decreases by 1 year to 72 over the transition if it shifts 3 years earlier, and by 2 years to age 71 if shifted 5 or 10 years earlier.

Consolidating these robustness checks, we feel the cut-off of 1871-1910 (as is reported in the main text) is an appropriate choice; though altering the cut-off may have slightly better model fits - and in some cases slightly different outcomes - the ‘best’ cut-off is not the same across each analysis.

Table S1. Comparison of models with different start and end points of the demographic transition for each type of analysis. The AIC of the lowest AIC models are bolded.

| Analysis | Transition period | AIC | ∆AIC^1^ | χ^2^ | | p | |
| --- | --- | --- | --- | --- | --- | --- | --- |
| Age at first birth | 1871-1910 | 11664 |  | 3.68 | | .720 | |
|  | 1870-1910 | **11660** | -4 | 2.90 | | .821 | |
|  | 1868-1910 | 11671 | 7 | 3.56 | | .736 | |
|  | 1866-1910 | 11680 | 16 | 3.64 | | .725 | |
|  | 1861-1910 | 11679 | 15 | 2.38 | | .882 | |
|  | 1871-1911 | 11664 | 0 | 3.55 | | .738 | |
|  | 1871-1913 | 11664 | 0 | 3.39 | | .758 | |
|  | 1871-1915 | 11664 | 0 | 3.35 | | .763 | |
|  | 1871-1920 | 11664 | 0 | 3.28 | | .773 | |
|  | 1870-1911 | **11660** | -4 | 4.08 | | .665 | |
|  | 1868-1913 | 11670 | 6 | 4.17 | | .653 | |
|  | 1866-1915 | 11680 | 16 | 2.65 | | .852 | |
|  | 1861-1920 | 11678 | 14 | 2.80 | | .834 | |
| Birth Spacing | 1871-1910 | **35871** |  | 14.09 | | .029 | |
|  | 1870-1910 | 35877 | 6 | 14.22 | | .027 | |
|  | 1868-1910 | 35883 | 12 | 14.39 | | .026 | |
|  | 1866-1910 | 35881 | 9 | 15.96 | | .014 | |
|  | 1861-1910 | 35881 | 9 | 16.47 | | .011 | |
|  | 1871-1911 | 35885 | 14 | 13.13 | | .041 | |
|  | 1871-1913 | 35892 | 21 | 13.14 | | .041 | |
|  | 1871-1915 | 35885 | 14 | 13.85 | | .031 | |
|  | 1871-1920 | 35896 | 25 | 13.79 | | .032 | |
|  | 1870-1911 | 35891 | 20 | 13.23 | | .039 | |
|  | 1868-1913 | 35902 | 31 | 13.35 | | .038 | |
|  | 1866-1915 | 35894 | 23 | 15.56 | | .016 | |
|  | 1861-1920 | 35908 | 37 | 14.74 | | .022 | |
| Survival | 1871-1910 | 22698 |  | 9.15 | | .01 | |
|  | 1870-1910 | **22665** | -33 | 10.01 | | .007 | |
|  | 1868-1910 | 22781 | 83 | 4.85 | | .089 | |
|  | 1866-1910 | 22781 | 83 | 7.31 | | .026 | |
|  | 1861-1910 | 22779 | 81 | 9.37 | | .009 | |
|  | 1871-1911 | 22705 | 7 | 9.46 | | .009 | |
|  | 1871-1913 | 22701 | 3 | 10.93 | | .004 | |
|  | 1871-1915 | 22705 | 7 | 10.88 | | .004 | |
|  | 1871-1920 | 22682 | -16 | 11.61 | | .003 | |
|  | 1870-1911 | 22672 | -26 | 10.32 | | .006 | |
|  | 1868-1913 | 22780 | 82 | 6.64 | | .036 | |
|  | 1866-1915 | 22790 | 92 | 8.89 | | .012 | |
|  | 1861-1920 | 22757 | 59 | 11.64 | | .003 | |
|  |  | Expected age at death from cumulative hazard^2^ | | |  | |  |
|  |  | Pre-industrial | Transitional | Post-transition | |  | |
| Hazard | 1871-1910 | 62 | 73 | 80 | |  | |
|  | 1870-1910 | 62 | 73 | 80 | |  | |
|  | 1868-1910 | 62 | 72 | 80 | |  | |
|  | 1866-1910 | 62 | 71 | 80 | |  | |
|  | 1861-1910 | 62 | 71 | 80 | |  | |
|  | 1871-1911 | 62 | 73 | 80 | |  | |
|  | 1871-1913 | 62 | 73 | 80 | |  | |
|  | 1871-1915 | 62 | 73 | 80 | |  | |
|  | 1871-1920 | 62 | 73 | 81 | |  | |
|  | 1870-1911 | 62 | 73 | 80 | |  | |
|  | 1868-1913 | 62 | 72 | 80 | |  | |
|  | 1866-1915 | 62 | 71 | 80 | |  | |
|  | 1861-1920 | 62 | 71 | 81 | |  | |

^1^ AIC relative to AIC of base model (transition 1871-1910), rather than relative to AIC of ‘best’ model

^2^ Age at which hazard is equal to 1


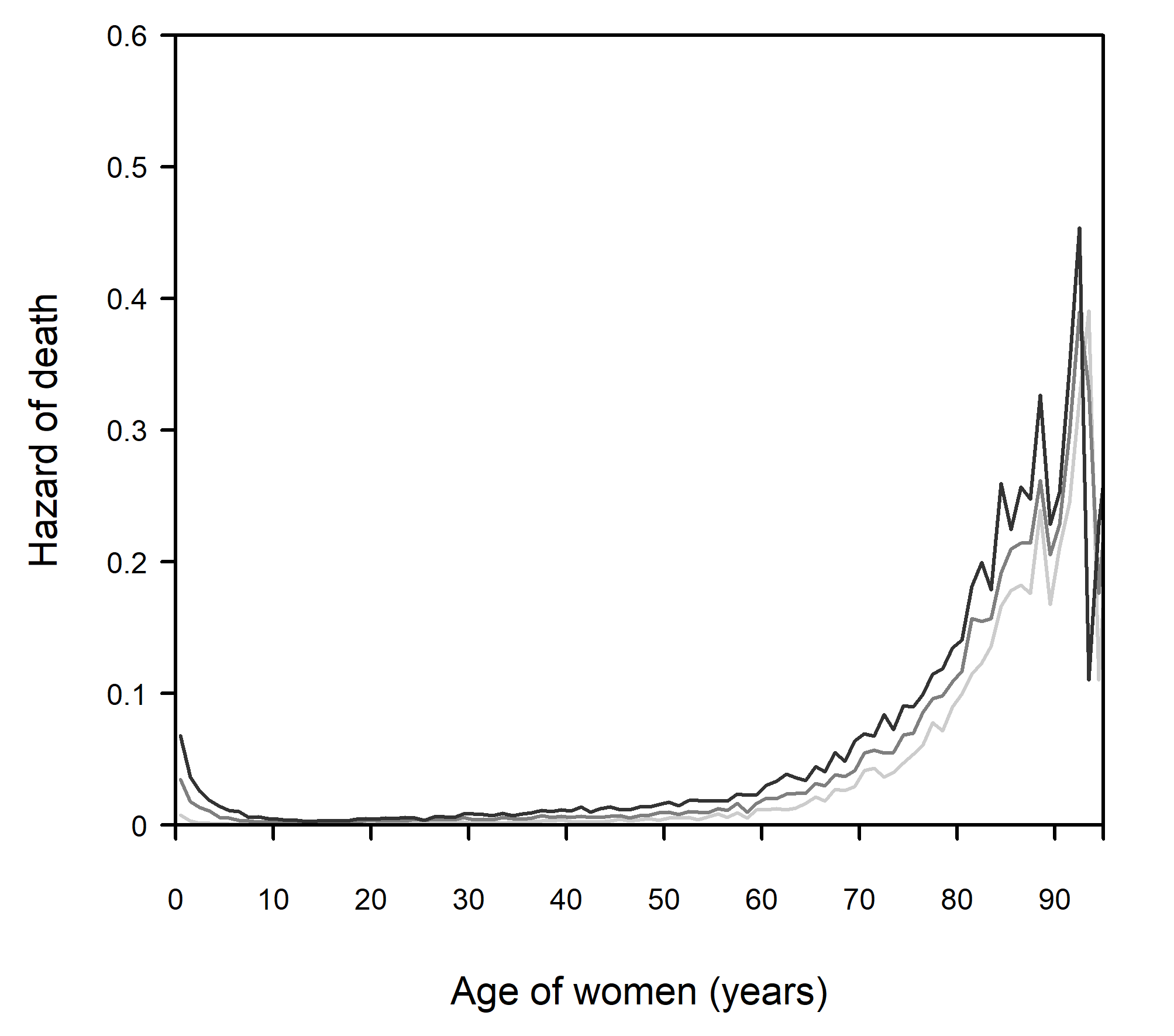


**Figure S1. Hazard functions for survival of women without censoring at the end of time periods, related to Figure 1.** Time periods pre-industrial (dark grey), transitional (medium grey), and post-transition (light grey) show similar hazard functions to Figure 1A. Later life hazard rates of transitional and pre-industrial periods are slightly lower, but still begin to increase earlier than in the post-transition period. From cumulative hazards, women would be expected to have died by ages 67 (pre-industrial), 75 (transitional), and 80 (post-transitional), a slight increase in age for the first two time periods compared to the hazards presented in the main text.

**Paternal grandmothers**

We ran the survival models with paternal grandmother presence instead of maternal grandmother presence to check whether the assumption that paternal grandmothers are not associated with grandchild survival (based on previous studies in this population) remained the case across the demographic transition and beyond. The interactions of paternal grandmother presence with either time period (χ^2^_3_ = 1.84, p = 0.398) or grandchild birth cohort (χ^2^_1_ = 0.07, p = 0.792) indicated that there were no changes over time in the (non-)association of paternal grandmother presence and grandchild survival. Coefficients in the time period model showed this to indeed be the case, with no effect pre-industrially (β = 0.942±0.739, p = 0.202), transitionally (β = 0.971±0.750, p = 0.196), or post-transitionally (β = -0.919±0.723, p = 0.204).

**Table S2.** **Generalised linear mixed-effect model outputs for grandchild survival by maternal grandmother presence, related to Figure 2**. MGM = maternal grandmother. MGM Reference levels for both models: MGM (dead), mother status (alive), singleton. In the ‘time period’ model, reference level was time period (post-transition).

| Model | Fixed effects | β ± standard error | z value | p value |
| --- | --- | --- | --- | --- |
| Time period | Intercept | 4.940 ± 0.271 | 18.24 | <0.001 |
|  | Age | 0.318 ± 0.018 | 17.32 | <0.001 |
|  | MGM (alive) | -0.228 ± 0.200 | -1.14 | 0.254 |
|  | Time period (pre-industrial) | -2.313 ± 0.249 | -9.28 | <0.001 |
|  | Time period (transition) | -1.195 ± 0.248 | -4.83 | <0.001 |
|  | Mother status (censored) | -0.540 ± 0.293 | -1.85 | 0.065 |
|  | Mother status (dead) | -0.778 ± 0.092 | -8.42 | <0.001 |
|  | Living siblings under age 16 | -0.032 ± 0.014 | -2.36 | 0.018 |
|  | Number of cousins | 0.015 ± 0.006 | 2.48 | 0.013 |
|  | Twin | -0.314 ± 0.119 | -2.65 | 0.008 |
|  | MGM (alive) x Time period (pre-industrial) | 0.514 ± 0.209 | 2.47 | 0.014 |
|  | MGM (alive) x Time period (transition) | 0.308 ± 0.209 | 1.47 | 0.141 |
| Birth cohort | Intercept | 1.622 ± 0.179 | 9.05 | <0.001 |
|  | Age | 0.332 ± 0.019 | 17.30 | <0.001 |
|  | MGM (alive) | 0.509 ± 0.151 | 3.37 | <0.001 |
|  | Birth cohort | 0.129 ± 0.009 | 13.77 | <0.001 |
|  | Mother status (censored) | -0.416 ± 0.312 | -1.33 | 0.183 |
|  | Mother status (dead) | -0.930 ± 0.099 | -9.41 | <0.001 |
|  | Living sibling under age 16 | -0.059 ± 0.015 | -3.97 | <0.001 |
|  | Number of cousins | 0.012 ± 0.007 | 1.71 | 0.088 |
|  | Twin | -0.272 ± 0.127 | -2.14 | 0.032 |
|  | MGM (alive) x Birth cohort | -0.026 ± 0.013 | -2.03 | 0.042 |
|  | Random effects | Variance | Standard deviation | |
| Time period | Mother ID:MGM ID | 0.134 | 0.366 | |
|  | MGM ID | 0.114 | 0.338 | |
|  | Birth cohort | 0.272 | 0.522 | |
| Birth cohort | Mother ID:MGM ID | 0.401 | 0.633 | |
|  | MGM ID | 0.192 | 0.438 | |

**Table S3.** **Generalised linear mixed-effect model outputs for offspring age at first birth and birth spacing by mother/mother-in-law presence, related to Figure 3**. MIL = mother-in-law, PI = pre-industrial, T = transition, TLB = time since last birth. Reference levels: Relation (dead), Time period (post-transition), Status of previous child (dead).

| Model | Fixed effects | β ± standard error | z value | p value |
| --- | --- | --- | --- | --- |
| Age at first birth  (time period) | Intercept | -15.900 ± 2.299 | -6.92 | <0.001 |
|  | Age | 0.992 ± 0.094 | 10.53 | <0.001 |
|  | Age (quadratic) | -0.015 ± 0.001 | -14.19 | <0.001 |
|  | Relation (both) | -1.439 ± 2.203 | -0.65 | 0.514 |
|  | Relation (mother) | -1.707 ± 2.455 | -0.70 | 0.487 |
|  | Relation (MIL) | -0.933 ± 2.437 | -0.38 | 0.702 |
|  | Time period (PI) | -1.433 ± 2.219 | -0.65 | 0.518 |
|  | Time period (T) | -2.120 ± 2.254 | -0.94 | 0.347 |
|  | Birth order | -0.024 ± 0.015 | -1.63 | 0.103 |
|  | Living siblings | -0.006 ± 0.039 | -0.16 | 0.871 |
|  | Age x Relation (both) | 0.041 ± 0.074 | 0.55 | 0.581 |
|  | Age x Relation (mother) | 0.049 ± 0.085 | 0.58 | 0.562 |
|  | Age x Relation (MIL) | -0.001 ± 0.083 | -0.01 | 0.992 |
|  | Age x Time period (PI) | 0.038 ± 0.074 | 0.52 | 0.605 |
|  | Age x Time period (T) | 0.072 ± 0.083 | 0.96 | 0.337 |
|  | Relation (both) x Time period (PI) | 1.122 ± 2.322 | 0.48 | 0.629 |
|  | Relation (mother) x Time period (PI) | 2.892 ± 2.588 | 1.12 | 0.264 |
|  | Relation (MIL) x Time period (PI) | 0.952 ± 2.586 | 0.37 | 0.713 |
|  | Relation (both) x Time period (T) | 2.243 ± 2.360 | 0.95 | 0.342 |
|  | Relation (mother) x Time period (T) | 3.442 ± 2.622 | 1.31 | 0.189 |
|  | Relation (MIL) x Time period (T) | 1.677 ± 2.633 | 0.64 | 0.524 |
|  | Age x Relation (both) x Time period (PI) | -0.047 ± 0.079 | -0.60 | 0.550 |
|  | Age x Relation (mother) x Time period (PI) | -0.106 ± 0.090 | -1.17 | 0.243 |
|  | Age x Relation (MIL) x Time period (PI) | -0.014 ± 0.090 | -0.16 | 0.873 |
|  | Age x Relation (both) x Time period (T) | -0.074 ± 0.081 | -0.92 | 0.357 |
|  | Age x Relation (mother) x Time period (T) | -0.113 ± 0.092 | -1.23 | 0.219 |
|  | Age x Relation (MIL) x Time period (T) | -0.037 ± 0.092 | -0.40 | 0.688 |
| Age at first birth  (birthing cohort) | Intercept | -16.160 ± 1.887 | -8.57 | <0.001 |
|  | Age | 1.021 ± 0.086 | 11.93 | <0.001 |
|  | Age (quadratic) | -0.015 ± 0.001 | -14.33 | <0.001 |
|  | Relation (both) | -1.541 ± 1.865 | -0.83 | 0.409 |
|  | Relation (mother) | 0.669 ± 1.991 | 0.34 | 0.737 |
|  | Relation (MIL) | -1.247 ± 2.107 | -0.59 | 0.554 |
|  | Birthing cohort | -0.157 ± 0.146 | -1.07 | 0.283 |
|  | Birth order | -0.014 ± 0.015 | -0.97 | 0.331 |
|  | Living siblings | -0.018 ± 0.039 | -0.45 | 0.651 |
|  | Age x Relation (both) | 0.013 ± 0.068 | 0.19 | 0.853 |
|  | Age x Relation (mother) | -0.062 ± 0.072 | -0.86 | 0.393 |
|  | Age x Relation (MIL) | 0.015 ± 0.077 | 0.20 | 0.845 |
|  | Age x Birthing cohort | 0.004 ± 0.005 | 0.70 | 0.482 |
|  | Relation (both) x Birthing cohort | 0.167 ± 0.158 | 1.06 | 0.289 |
|  | Relation (mother) x Birthing cohort | 0.069 ± 0.170 | 0.41 | 0.684 |
|  | Relation (MIL) x Birthing cohort | 0.152 ± 0.178 | 0.85 | 0.394 |
|  | Age x Relation (both) x Birthing cohort | -0.003 ± 0.006 | -0.58 | 0.562 |
|  | Age x Relation (mother) x Birthing cohort | <0.001 ± 0.006 | 0.02 | 0.980 |
|  | Age x Relation (MIL) x Birthing cohort | -0.004 ± 0.007 | -0.64 | 0.524 |
| Birth spacing | Intercept | 2.086 ± 0.230 | 9.06 | <0.001 |
|  | Age | -0.107 ± 0.004 | -26.14 | <0.001 |
|  | TLB | -0.193 ± 0.041 | -4.70 | <0.001 |
|  | Relation (both) | -0.550 ± 0.235 | -2.34 | 0.019 |
|  | Relation (mother) | -0.132 ± 0.252 | -0.53 | 0.600 |
|  | Relation (MIL) | -0.320 ± 0.272 | -1.18 | 0.240 |
|  | Time period (PI) | -0.049 ± 0.222 | -0.22 | 0.827 |
|  | Time period (T) | 0.214 ± 0.216 | 0.99 | 0.321 |
|  | Status of previous child (alive) | -0.190 ± 0.048 | -3.97 | <0.001 |
|  | Next child birth order | 0.055 ± 0.011 | 5.17 | <0.001 |
|  | Living siblings | 0.087 ± 0.018 | 4.84 | <0.001 |
|  | TLB x Relation (both) | 0.142 ± 0.049 | 2.91 | 0.003 |
|  | TLB x Relation (mother) | 0.040 ± 0.051 | 0.77 | 0.440 |
|  | TLB x Relation (MIL) | 0.047 ± 0.057 | 0.82 | 0.412 |
|  | TLB x Time period (PI) | 0.112 ± 0.044 | 2.52 | 0.012 |
|  | TLB x Time period (T) | 0.106 ± 0.043 | 2.47 | 0.014 |
|  | Relation (both) x Time period (PI) | 0.020 ± 0.257 | 0.08 | 0.939 |
|  | Relation (mother) x Time period (PI) | -0.024 ± 0.273 | -0.09 | 0.930 |
|  | Relation (MIL) x Time period (PI) | 0.221 ± 0.295 | 0.75 | 0.454 |
|  | Relation (both) x Time period (T) | -0.206 ± 0.251 | -0.82 | 0.412 |
|  | Relation (mother) x Time period (T) | -0.236 ± 0.267 | -0.88 | 0.378 |
|  | Relation (MIL) x Time period (T) | -0.003 ± 0.289 | -0.01 | 0.991 |
|  | TLB x Status of previous child (alive) | 0.094 ± 0.013 | 7.35 | <0.001 |
|  | TLB x Relation (both) x Time period (PI) | 0.180 ± 0.057 | 3.17 | 0.002 |
|  | TLB x Relation (mother) x Time period (PI) | 0.110 ± 0.058 | 1.92 | 0.056 |
|  | TLB x Relation (MIL) x Time period (PI) | 0.062 ± 0.063 | 0.98 | 0.329 |
|  | TLB x Relation (both) x Time period (T) | 0.133 ± 0.055 | 2.43 | 0.015 |
|  | TLB x Relation (mother) x Time period (T) | 0.09 1± 0.056 | 1.62 | 0.105 |
|  | TLB x Relation (MIL) x Time period (T) | 0.111 ± 0.062 | 1.79 | 0.074 |
|  | Random effects | Variance | Standard deviation | |
| Age at first birth | Mother ID | 0.768 | 0.877 | |
| (time period) |  |  |  | |
| Age at first birth | Mother ID | 0.814 | 0.902 | |
| (birthing cohort) |  |  |  | |
| Birth spacing | ID:Mother ID | 0.192 | 0.438 | |
|  | Mother ID | 0.073 | 0.269 | |
